# Bone cancer induces a unique central sensitization through synaptic changes in a wide area of the spinal cord

**DOI:** 10.1186/1744-8069-6-38

**Published:** 2010-07-05

**Authors:** Yoshikazu Yanagisawa, Hidemasa Furue, Tomoyuki Kawamata, Daisuke Uta, Jun Yamamoto, Shingo Furuse, Toshihiko Katafuchi, Keiji Imoto, Yukihide Iwamoto, Megumu Yoshimura

**Affiliations:** 1 Department of Integrative Physiology, Graduate School of Medical Sciences, Kyushu University, Fukuoka, Japan; 2 Department of Orthopedic Surgery, Kyushu University, Fukuoka, Japan; 3 Department of Information Physiology, National Institute for Physiological Sciences, Okazaki, Japan; 4 Department of Anesthesiology, Sapporo Medical University, Sapporo, Japan; 5 Department of Anesthesiology and Resuscitology, Shinshu University School of Medicine, Matsumoto, Japan; 6 Graduate School of Health Sciences, Kumamoto Health Science University, Kumamoto, Japan

## Abstract

**Background:**

Chronic bone cancer pain is thought to be partly due to central sensitization. Although murine models of bone cancer pain revealed significant neurochemical changes in the spinal cord, it is not known whether this produces functional alterations in spinal sensory synaptic transmission. In this study, we examined excitatory synaptic responses evoked in substantia gelatinosa (SG, lamina II) neurons in spinal cord slices of adult mice bearing bone cancer, using whole-cell voltage-clamp recording techniques.

**Results:**

Mice at 14 to 21 days after sarcoma implantation into the femur exhibited hyperalgesia to mechanical stimuli applied to the skin of the ipsilateral hind paw, as well as showing spontaneous and movement evoked pain-related behaviors. SG neurons exhibited spontaneous excitatory postsynaptic currents (EPSCs). The amplitudes of spontaneous EPSCs were significantly larger in cancer-bearing than control mice without any changes in passive membrane properties of SG neurons. In the presence of TTX, the amplitude of miniature EPSCs in SG neurons was increased in cancer-bearing mice and this was observed for cells sampled across a wide range of lumbar segmental levels. Alpha-amino-3-hydroxy-5-methyl-4-isoxazolepropionic acid (AMPA) receptor- and *N*-methyl-*D*-aspartate (NMDA) receptor-mediated EPSCs evoked by focal stimulation were also enhanced in cancer-bearing mice. Dorsal root stimulation elicited mono- and/or polysynaptic EPSCs that were caused by the activation of Aδ and/or C afferent fibers in SG neurons from both groups of animals. The number of cells receiving monosynaptic inputs from Aδ and C fibers was not different between the two groups. However, the amplitude of the monosynaptic C fiber-evoked EPSCs and the number of SG neurons receiving polysynaptic inputs from Aδ and C fibers were increased in cancer-bearing mice.

**Conclusions:**

These results show that spinal synaptic transmission mediated through Aδ and C fibers is enhanced in the SG across a wide area of lumbar levels following sarcoma implantation in the femur. This widespread spinal sensitization may be one of the underlying mechanisms for the development of chronic bone cancer pain.

## Background

Pain is one of the most common symptoms of patients with primary bone sarcomas and those with bone metastases from lung or other solid tumors [1,2]. It is a troublesome clinical problem since current treatments are not fully effective. As the disease progresses, chronic pain develops and often becomes resistant to conventional analgesic treatments such as anti-inflammatory drugs and opioids even at the maximum doses that can be tolerated [2,3]. Despite the global efforts designed to treat both bone cancer and cancer-related pain, nearly 70% of the patients dying from cancer experience unrelieved pain which is usually progressive and severe [4].

In recent years, preclinical animal models of bone cancer pain have been developed and the mechanisms contributing to bone cancer pain have been studied at cellular and behavioral levels [5-8]. Implantation of cancer cells within the femur or tibia induces ongoing pain-related behaviors such as licking and flinching of the ipsilateral hind limb following bone destruction [9-12] associated with injuries of the primary afferent fibers innervating the cancer-bearing bones [12]. In addition to these spontaneous behavioral changes, previous studies show that animals bearing bone cancer also exhibit allodynia and/or hyperalgesia in response to natural sensory stimuli applied to the non-cancerous skin [9,12,13]. In addition to sensitization of afferent fibers in animals bearing bone cancer [5], the excitability (firing rates) of spinal dorsal horn neurons in response to cutaneous sensory stimuli is reported to be increased [8,14] along with an enlargement of their receptive field sizes [8]. These functional changes in responsiveness of spinal dorsal horn neurons may be induced not only by peripheral sensitization of afferent fibers innervating the cancerous bone, but also by processes of spinal sensitization, such as plastic changes in spinal noxious circuitry or in synaptic transmission as has been reported in other animal models of peripheral nerve injuries and inflammation [15]. Extensive neurochemical reorganization and gliosis in the spinal dorsal horn of animals bearing bone cancer support the hypothesis that bone cancer pain is enhanced by a state of spinal sensitization [6,7,13,16]. However little is known about the functional changes in the synaptic processing of nociceptive neuronal circuitry of the spinal dorsal horn in animals bearing bone cancer.

The substantia gelatinosa (SG, lamina II) of the spinal dorsal horn plays an important role in the transmission and modulation of nociceptive information from the periphery to the CNS [17-20], and is one of the key sites for the generation of central sensitization in chronic neuropathic pain states [15]. Fine myelinated Aδ and unmyelinated C afferent fibers terminate preferentially in the SG. SG neurons exhibit glutamatergic excitatory postsynaptic responses in response to stimulation of Aδ and C afferent fibers [19,21-23] and naturalistic noxious stimulation [24-26]. Following inflammation and peripheral nerve injuries, the excitatory sensory pathways in the SG are functionally reorganized: large fine myelinated Aβ fibers establish aberrant synaptic contacts with SG neurons [27-29]. The present study was designed to assess whether excitatory sensory pathways and synaptic transmission in the SG are changed in adult mice bearing bone cancer using patch-clamp recordings from spinal cord slices with an attached dorsal root. We compared excitatory synaptic responses evoked in SG neurons between control mice and mice bearing cancer in the femur, and show that the cancer-bearing mice exhibit unique plastic changes in spinal excitatory synaptic transmission mediated through Aδ and C afferent fibers.

## Results

### Spontaneous and movement-evoked pain-related behaviors and hypersensitivity to mechanical stimuli in mice with bone cancer

**Figure 1:**
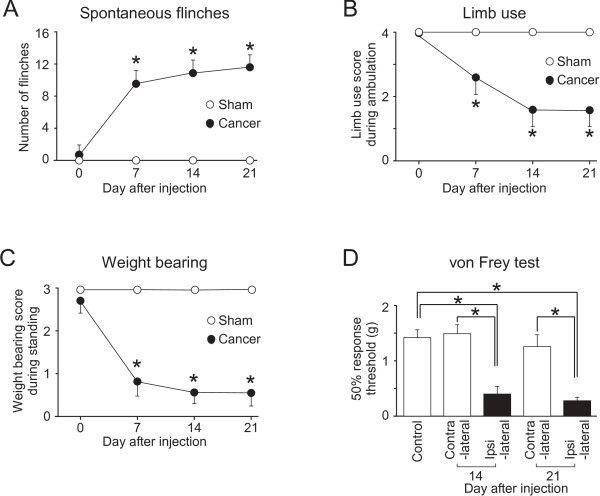
**Spontaneous and movement evoked pain-related behaviors and mechanical hyperalgesia following sarcoma implantation in the femur**. Time course of the number of spontaneous flinches of the cancer-bearing hind limb (A), unimpaired limb use score during spontaneous ambulation (B) and weight bearing score during standing (C) following sarcoma implantation (n = 6 for each test, closed circles). *Significantly different from the values obtained from sham-operated mice (n = 6 for each test, open squares), *P *< 0.05 (the Kruskal-Wallis test followed by the Scheffe test). Withdrawal thresholds to von Frey filament stimulation to the hind paw skin ipsilateral to cancer-bearing femur at 14 and 21 days after sarcoma implantation (n = 14) (D). *Significantly different from the values for the stimulation applied to the contralateral paw of cancer-bearing mice (n = 14) and those obtained from control mice (n = 20), *P *< 0.05 (non-repeated measures ANOVA by post hoc test or Dunnett's test). Values represent means ± S.E.M.

Mice had unilateral implantation of osteolytic murine sarcoma cells into the femur [7,30]. After sarcoma implantation, the animals exhibited spontaneous foot-lifting behavior of the cancer-bearing limb. The number of spontaneous flinches was significantly increased at day 7 after implantation, and the increment lasted for more than 2 weeks (Figure 1A). Cancer-bearing mice also exhibited impaired limb use during spontaneous ambulation. The limb use score gradually decreased after sarcoma implantation to reach a stable nadir at days 14 to 21 after sarcoma implantation (Figure 1B). A lower weight-bearing score during standing was also observed in these mice at days 7 to 21 after sarcoma implantation (Figure 1C). In contrast, sham-operated mice which received an injection of culture medium without sarcoma cells in the femur, had no spontaneous flinches and showed no change in their limb use and weight-bearing scores (Figures 1A-C).

Next we examined the mechanical sensitivity of the hind paw at days 14 and 21 after sarcoma implantation. The paw withdrawal thresholds of the ipsilateral hind paw (assessed with von Frey filaments) were significantly lower than those on the contralateral side and also those in control mice (Figure 1D). The values of the paw withdrawal thresholds in sham-operated mice were similar to those in control mice (data not shown). These results suggest that the hindpaw of the cancer bearing limb has developed hypersensitivity to mechanical stimuli applied to the skin. Based on these initial findings we progressed to search for electrophysiological evidence of changes in spinal nociceptive processing in slice preparations of the spinal cord obtained from mice 14 to 21 days after sarcoma implantation. The excitatory synaptic responses in the SG ipsilateral to the cancer-bearing side were compared with those in control mice.

### Increased amplitudes of spontaneous and miniature EPSCs in SG neurons of bone cancer-bearing mice

In the behavioral analysis, cancer-bearing mice exhibited hyperalgesia in response to mechanical stimulation applied to the hind paw. The hind paw afferent innervation synapses with second order neurons in the lumbar spinal segments (mainly in L3-5) [31,32]. Therefore, we first examined the passive membrane properties and the synaptic inputs to SG neurons in the L3-5 segments. All SG neurons examined had resting membrane potentials more negative than -55 mV in control and cancer-bearing mice. No differences were found in the resting membrane potential (control, -65.3 ± 1.0 mV, n = 26; cancer, -65.7 ± 0.8 mV, n = 34; *P *= 0.98) and input membrane resistance (control, 625.9 ± 62.8 MΩ, n = 26; cancer, 714.4 ± 34.5 MΩ, n = 34; *P *= 0.18) between the two groups.

**Figure 2:**
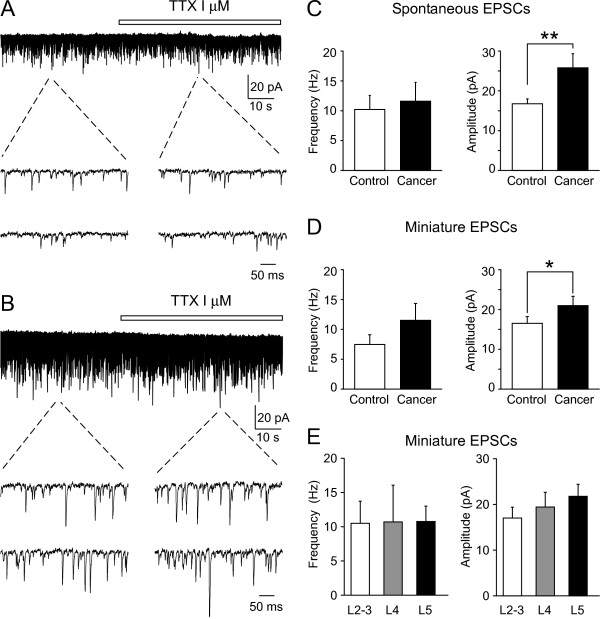
**Spontaneous and miniature EPSCs evoked in SG neurons at lumbar spinal levels in control and cancer-bearing mice**. Representative traces of sEPSCs and mEPSCs in control (A) and cancer-bearing mice (B). Horizontal bars given above the traces show the duration of drug application. Two lower records in *A *and *B*: consecutive traces of EPSCs in the absence (*right*) and presence (*left*) of TTX shown in an expanded scale in time. The frequency (*left*) and amplitude (*right*) of sEPSCs (C) and mEPSCs (D) in SG neurons at spinal levels of L3-5. The amplitudes of sEPSCs and mEPSCs were significantly larger in cancer-bearing than control mice, *P < 0.05 (unpaired t-test). The frequency and amplitude of mEPSCs at different lumber levels (L2-3, n = 5; L4, n = 6; L5, n = 12) in cancer-bearing mice (E). There were no significant differences in the frequency and amplitude of mEPSCs between the three groups. Values represent means ± S.E.M.

SG neurons in control and cancer-bearing mice exhibited spontaneous EPSCs (sEPSCs), recorded under voltage-clamp at a holding potential of -70 mV, (Figures 2A, B). The frequency of sEPSCs was not significantly different between the two groups (control, 10.4 ± 2.5 Hz, n = 18; cancer, 11.8 ± 3.2 Hz, n = 11; *P *= 0.36). The amplitude of sEPSC, on the other hand, was significantly larger in cancer-bearing than control mice (control, 17.0 ± 1.5 pA, n = 18; cancer, 25.7 ± 3.7 pA, n = 11; *P *< 0.01) (Figure 2C). In the presence of TTX, the amplitude of miniature EPSC (mEPSCs) was still significantly larger in cancer-bearing than control mice (control, 16.8 ± 1.6 pA, n = 18; cancer, 22.3 ± 2.6 pA, n = 11; *P *< 0.05) (Figures 2A, B, D). The mEPSC frequency was not significantly different between the two groups (control, 7.5 ± 1.9 Hz, n = 18; cancer, 11.5 ± 3.1 Hz, n = 11, *P *= 0.12). These results suggest that there is a facilitation of excitatory synaptic transmission onto SG neurons at lumbar levels in mice bearing cancer in the femur, but that there is no alteration in their passive membrane properties.

The femur, the bone bearing cancer in this study, is innervated by the femoral nerve which has its central terminations mainly in the upper lumbar levels of spinal cord (L2-3) [12]. By contrast, the hind paw, which shows mechanical hyperalgesia, is innervated by the sciatic nerve which terminates mainly in lumbar segments L4-5 [31,32]. We further compared the amplitude and frequency of mEPSCs in cancer-bearing mice among three groups of SG neurons which were in different lumbar levels: SG neurons in spinal cord slices at lumbar levels of L2-3, L4 or L5. However, there were no significant differences in the frequency (L2-3, 10.4 ± 3.1 Hz, n = 5; L4, 10.6 ± 5.3 Hz, n = 6; L5, 10.7 ± 2.1 Hz, n = 12; *P *= 0.72) and amplitude (L2-3, 16.8 ± 2.3 pA, n = 5; L4, 19.3 ± 3.2 pA, n = 6; L5, 21.7 ± 2.6 pA, n = 12; *P *= 0.36) of mEPSCs among the three groups in cancer-bearing mice (Figure 2E). These results suggest that increased excitatory synaptic responses in the SG are not restricted to the area of central terminations of the afferent fibers innervating cancerous bones, and are detected throughout wide area of lumbar levels. To explore this further we examined synaptic responses elicited in SG neurons at the lumbar levels of L4-5 whose dermatomes include the hind paw.

### Increased AMPA-induced currents and evoked EPSCs in bone cancer-bearing mice

**Figure 3:**
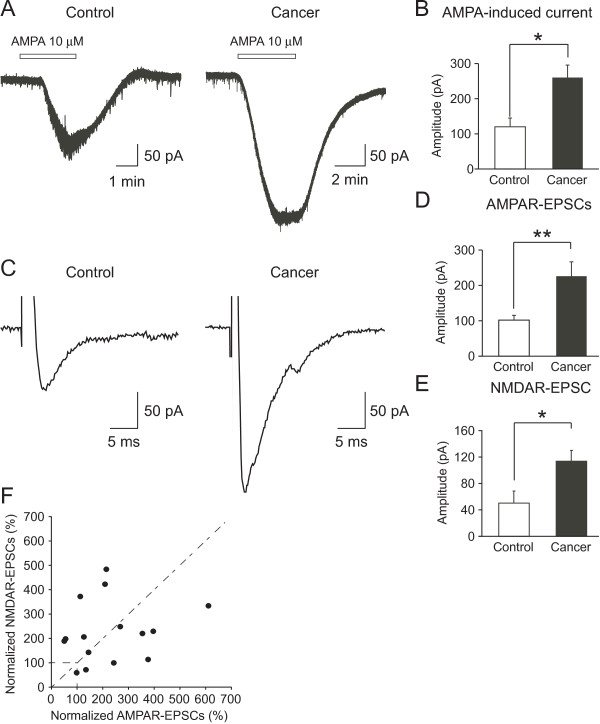
**AMPA-induced inward currents and focally evoked EPSCs in SG neurons of control and cancer-bearing mice**. Representative traces of inward currents induced by bath-application of AMPA (10 μM) in control (*left*) and cancer-bearing mice (*right*) (A). The amplitude of AMPA-induced inward currents was significantly greater in cancer-bearing (n = 12) than control mice (n = 14), *P < 0.05 (unpaired t-test) (B). Representative averaged traces of EPSCs evoked by focal stimulation through an electrode positioned near the recorded neurons in control (*left*) and cancer-bearing (*right*) mice (C). The amplitude of the focal stimulation-evoked (AMPAR-) EPSCs was significantly larger in cancer-bearing mice (n = 7) than control mice (n = 15), *P < 0.05 (unpaired t-test) (D). The amplitude of the focal stimulation-evoked (NMDAR-) EPSCs was also significantly greater in cancer-bearing mice (n = 7) than control mice (n = 15), *P < 0.05 (unpaired t-test) (E). Relationship between the amplitudes of the focal AMPAR- and NMDAR-EPSCs evoked in single SG neurons of cancer-bearing mice (F). The amplitudes of the focal NMDAR- and AMPAR-EPSCs evoked in the same SG neurons of cancer-bearing mice were normalized to the average amplitudes of NMDAR- and AMPAR-EPSCs of control mice, respectively, and then the normalized amplitudes of NMDAR-EPSCs were plotted against those of AMPAR-EPSCs. The correlation coefficient of the linear regression for the normalized amplitudes of NMDAR- versus AMPAR-EPSCs was 0.20 (Linear regression; y = 0.17 × + 188.32, *P *> 0.05). Values represent means ± S.E.M.

In order to determine whether the increased amplitude of mEPSCs in cancer-bearing mice was postsynaptic in origin, we compared AMPA-induced currents elicited in postsynaptic SG neurons between the two groups. At a holding potential of -70 mV, application of AMPA (10 μM) elicited an inward current in all SG neurons tested in control and cancer-bearing mice. The peak amplitude of the AMPA-induced current was significantly larger in cancer-bearing than control mice (control, 127.2 ± 21.5 pA, n = 12; cancer, 260.0 ± 35.0 pA, n = 23; *P *< 0.01) (Figures 3A, B). The times to peak of the AMPA response in control and cancer-bearing mice were 1.80 ± 0.27 min (n = 12) and 1.53 ± 0.22 min (n = 23), respectively. There was no significant difference between the groups (*P *> 0.05, unpaired t-test).

Next we examined whether evoked synaptic responses mediated by AMPA receptors were also enhanced in cancer-bearing mice. In the presence of a GABA_A _receptor antagonist bicuculline (10 μM) and a glycine receptor antagonist strychnine (2 μM), a monopolar stimulating electrode was placed near the recorded SG neuron, and EPSCs evoked by the focal stimulation were compared between the two groups (at a holding potential of -70 mV). The focally evoked EPSCs were completely inhibited by supplemental addition of CNQX (10 μM) (control, n = 7; cancer, n = 15). The amplitude of focal AMPA receptor-mediated EPSCs (focal AMPAR-EPSCs) was significantly larger in cancer-bearing than control mice (control, 101.1 ± 13.7 pA, n = 7; cancer, 226.8 ± 40.1 pA, n = 15; *P *< 0.05) (Figures 3C, D). We further examined the focally evoked EPSCs mediated by NMDA receptors (focal NMDAR-EPSCs) in the two groups, in the presence of CNQX (10 μM), bicuculline (10 μM) and strychnine (2 μM) at a holding potential of +40 mV (to reveal the NMDA mediated component). The focal NMDAR-EPSCs had a long duration (~400 ms), and the responses were completely suppressed by supplemental addition of APV (50 μM; control, n = 2; cancer, n = 2). The amplitude of focal NMDAR-EPSCs was also significantly increased in cancer-bearing mice (control, 51.6 ± 18.4 pA, n = 7; cancer, 116.4 ± 16.9 pA, n = 15; *P *< 0.05) (Figure 3E). There was little correlation between the normalized focal NMDAR- and AMPAR-EPSCs elicited in individual SG neurons of cancer-bearing mice (*r *= 0.20) (Figure 3F). This suggests that the AMPA and NMDA receptor-mediated EPSCs in individual SG neurons are not concordantly increased and also that the increased amplitudes of the miniature and evoked responses of SG neurons in cancer-bearing mice are unlikely to be due simply to an increase in presynaptic glutamate release.

### Plastic changes in spinal sensory pathways to SG neurons from afferent fibers in bone cancer-bearing mice

**Figure 4:**
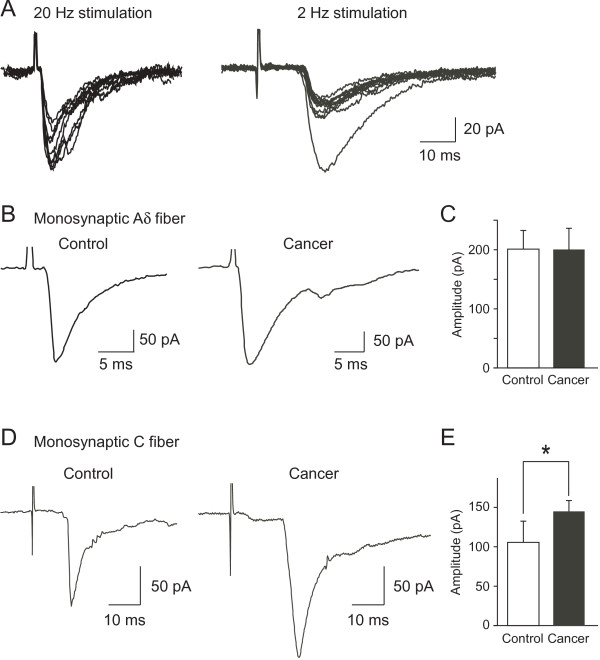
**Monosynaptic afferent fiber-evoked EPSCs in SG neurons of control and cancer-bearing mice**. Monosynaptic Aδ and C fiber-evoked EPSCs in response to the stimulation of dorsal root attached to the spinal cord slices (A). The monosynaptic nature was clarified based on their constant latencies and absence of failures with repetitive stimulations (20 Hz and 2 Hz for Aδ and C fiber's responses, respectively; see *Methods*). Representative averaged traces of monosynaptic Aδ fiber-evoked EPSCs in control (*left*) and cancer-bearing (*right*) mice (B). There was no significant difference in the amplitude of monosynaptic Aδ fiber-evoked EPSCs between control (n = 13) and cancer-bearing (n = 14) mice (*right*) (C). Representative averaged traces of C fiber-evoked EPSCs in control (*left*) and cancer-bearing (*right*) mice (D). The amplitude of monosynaptic C fiber-evoked EPSCs was significantly increased in cancer-bearing (n = 12) than control mice (n = 10), *P *< 0.05 (Mann-Whitney U-test) (E). Values represent means ± S.E.M.

**Figure 5:**
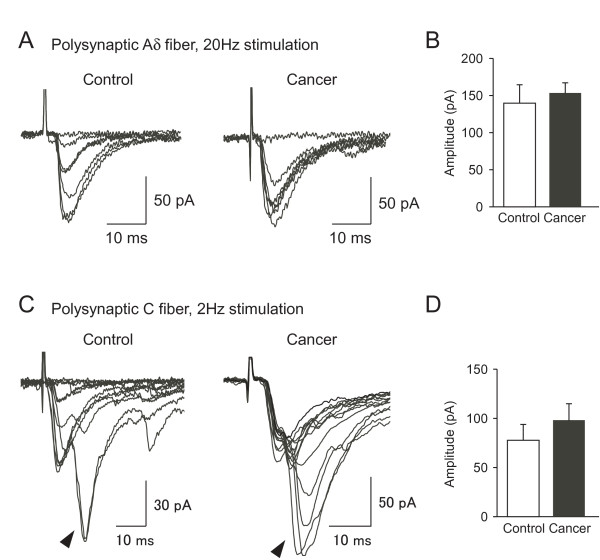
**Polysynaptic afferent fiber-evoked EPSCs in SG neurons of control and cancer-bearing mice**. Representative traces of polysynaptic Aδ fiber-evoked EPSCs elicited by the stimulation of the dorsal root at 20 Hz in control (*left*) and cancer-bearing mice (*right*) (A). There was no significant difference in the amplitude of polysynaptic Aδ fiber-evoked EPSCs between control (n = 26) and cancer-bearing (n = 34) mice (B). Representative traces of polysynaptic C fiber-evoked EPSCs (indicated by arrowheads) elicited by the stimulation of the dorsal root at 2 Hz in control (*left*) and cancer-bearing (*right*) mice (C). The neurons shown in *C *received polysynaptic C fiber-evoked EPSCs and Aδ fiber-evoked ones. There was no significant difference in the amplitude of polysynaptic C fiber-evoked EPSCs between control (n = 8) and (n = 14) cancer-bearing mice, *P *> 0.05 (unpaired t-test) (D). Values represent means ± S.E.M.

Previous studies have shown that spinal sensory pathways to SG neurons from afferent fibers are drastically changed in other neuropathic pain models such as inflammation or peripheral nerve injuries [27-29]. To evaluate whether the excitatory afferent synaptic inputs to SG neurons were also changed in cancer-bearing mice, EPSCs evoked by stimulation of the dorsal root attached to the spinal cord slices were compared between the two groups (Figure 4A). In control and cancer-bearing mice, stimulation of the dorsal root evoked monosynaptic and/or polysynaptic EPSCs in 48 out of 228 (21%) and 62 out of 160 (39%) SG neurons, respectively. The dorsal root-evoked EPSCs could be classified into two groups based on the conduction velocities and stimulus intensities (Aδ and C afferent fiber-evoked responses, see METHODS). In control mice, monosynaptic Aδ and C fiber-evoked EPSCs were detected in 13 and 12 SG neurons, respectively, with an average conduction velocity of 3.50 ± 0.34 and 0.63 ± 0.04 m/s. Aδ and C fiber-evoked EPSCs in cancer-bearing mice were detected in 14 and 10 SG neurons, respectively, with an average conduction velocity of 3.00 ± 0.20 and 0.64 ± 0.04 m/s. The values of conduction velocities for Aδ and C fiber-evoked EPSCs were consistent with those obtained from SG neurons of rats [27-29]. There was no significant difference in the numbers of SG neurons receiving the monosynaptic EPSCs from Aδ or C fibers between the two groups (Table 1, χ^2^-test, *P *> 0.05) (Table 1). The amplitude of monosynaptic Aδ fiber-evoked EPSCs was not changed across groups (control, 199.0 ± 32.8 pA, n = 13; cancer, 199.6 ± 37.6 pA, n = 14; *P *= 0.49) (Figures 4B, C). In contrast, the amplitude of monosynaptic C fiber-evoked EPSCs was significantly increased in cancer-bearing mice relative to that in control mice (control, 106.6 ± 31.7 pA, n = 12; cancer, 147.3 ±13.8 pA, n = 10; *P *< 0.05) (Figures 4D, E). Furthermore, the proportion of SG neurons receiving polysynaptic Aδ or C fiber-evoked EPSCs was increased in cancer-bearing mice (Table 1, χ^2^-test, *P *< 0.05). There were no changes in the amplitude of polysynaptic Aδ fiber-evoked EPSCs (control, 139.3 ± 23.7 pA, n = 26; cancer, 150.2 ± 14.0 pA, n = 34, *P *= 0.33) nor in C fiber-evoked EPSCs (control, 78.1 ± 16.3 pA, n = 8; cancer, 99.3 ± 18.1 pA, n = 14; *P *= 0.22) between the two groups (Figure 5). These results suggest that in addition to the enhanced monosynaptic responses mediated through C fibers, the numbers of SG neurons receiving excitatory polysynaptic inputs from Aδ and C fibers are also increased in cancer-bearing mice.

**Table 1:** Number of SG neurons receiving monosynaptic and polysynaptic inputs in control and bone cancer-bearing mice

		Aδ fiber	C fiber	No input
Monosynaptic	Control	13 (6%)	12 (5%)	203 (89%)
	Cancer	14 (9%)	10 (6%)	136 (85%)
Polysynaptic	Control	26 (11%)	8 (4%)	194 (85%)
	Cancer	34 (21%)	14 (9%)	112 (70%)

The χ^2^-test revealed a significant difference in the number of cells receiving polysynaptic inputs from Aδ and C fibers between control and bone cancer-bearing mice (*P *< 0.05). The proportion of cells receiving monosynaptic inputs was not different across the groups.

## Discussion

The present study demonstrates that excitatory synaptic transmission is functionally enhanced in the SG throughout wide area of lumbar spinal levels in adult bone cancer-bearing mice. Furthermore we show that sensory pathways to SG neurons from Aδ and C fibers are also plastically altered. These widespread neural changes are likely to contribute to the "referred" hyperalgesia that develops in the hindpaw as the sarcoma grows in the femur. The present results indicate that bone cancer causes a unique spinal sensitization in noxious circuitry that is different from that induced by other neuropathic models such as peripheral nerve injuries and inflammation [15,27-29]. However these functional changes are compatible with the previous studies of mice with femoral bone cancer showing spinal neurochemical reorganizations in multiple lumbar segments of the spinal cord [7,13,16,33].

### Behavioral changes and plastic alterations in a large area of the spinal cord following cancer cell implantation in the femur

To study the pathophysiology of cancer pain, several animal models of bone cancer pain have been developed for rodents [5-8]. We used the bone cancer model of implanting NCTC2472 osteolytic sarcoma cells, because this model is reproducible and most widely used [7,9,11,13,34-36]. After sarcoma implantation into the medullary space of the femur, the cancer-bearing mice started showing pain-related behaviors at 7 to 21 days (Figures 1A-C). These previous studies also reported stereotyped, pain-related behaviors, which included flinching and limited limb use [9-12]. Interestingly, behavioral changes suggesting hypersensitivity of the hind paw of bone cancer models have been reported [9,12,13]. The finding of lowered withdrawal threshold for mechanical stimuli applied to the paw was confirmed in our study (Figure 1D). These findings suggest that bone cancer may induce a spinal sensitization over a wider area of the spinal cord than just the segments in which the central terminals of afferent fibers innervating the cancer-bearing tissues are located. Specifically the spinal segments receiving afferent fibers from the cancerous femur and the hind paw, are relatively distinct (L1-L3 for the femur; L3-5 for the paw) [12]. This notion is supported by the observation that c-Fos expression is upregulated in L3-L5, and GFAP labeling is increased in L2-L5 in animals bearing cancer in the femur [7]. An increased astrogliosis in the spinal cord segments of T10-S1 has been also shown in mice bearing cancer in the femur [13]. The present study is the first study demonstrating functional enhancement of excitatory synaptic transmission in the SG over a similarly wide area of lumbar levels (L2-5) in mice bearing cancer in the femur (Figure 2).

### Increased spinal excitatory synaptic responses in bone cancer model

To examine whether there was a change at the gateway of the spinal sensory pathways in cancer-bearing mice, we first compared the frequency and amplitude of sEPSCs and mEPSCs in SG neurons. We found that the amplitude of sEPSCs and mEPSCs were significantly increased, whereas their frequencies were unchanged (Figure 2). These results contrast with other neuropathic pain models, in which the amplitude and frequency of sEPSCs of SG neurons are unaffected in peripheral nerve injury models [27,29]. The increased amplitude and unchanged frequency in cancer-bearing mice suggested a postsynaptic mechanism is operative to increase the synaptic responses in SG neurons. To confirm this notion, we measured the amplitude of inward currents elicited in SG neurons by application of AMPA. This finding of an increase in the amplitude of the AMPA-induced current in cancer-bearing mice supports the postsynaptic mechanism of enhancement (Figures 3A, B). An alternative mechanism would posit that the increased EPSC amplitude could result from an increased amount of presynaptic glutamate release. Therefore, we measured NMDAR and AMPAR-EPSCs in the same neurons of cancer-bearing mice, and showed that the amplitude of both EPSCs was increased in cancer-bearing mice, but the increases of NMDAR- and AMPAR-EPSCs in single SG neurons were not correlated (Figure 3F), again supporting the postsynaptic mechanism. Based on these results, we conclude that the increased amplitudes of excitatory synaptic responses were mainly postsynaptic in origin, although the possibility of presynaptic changes cannot be completely ruled out.

In the present study, we did not identify how postsynaptic responses mediated by AMPA and NMDA receptors were increased. In inflamed animals, the subunit composition of AMPA receptors expressed in SG neurons is reported to be changed [37]. Such changes in subunit composition of AMPA receptors are known to alter the kinetics of the synaptic currents [38]. We compared the kinetics of the evoked EPSCs (decay time to 34% of the response) between control and cancer-bearing mice. The decay time constants of monosynaptic C fiber-evoked EPSCs in control and model mice were 9.2 ± 1.9 ms (n = 7) and 8.0 ± 1.1 ms (n = 7), respectively. There is no significant difference between the two groups (*P *> 0.05, unpaired t-test). Those for monosynaptic Aδ fiber-evoked EPSCs also did not differ between the two groups (control, 6.3 ± 1.0 ms, n = 9; model, 4.7 ± 0.9 ms, n = 8; *P *> 0.05, unpaired t-test). These results suggest that the increased responses in the bone cancer model mice may be due to increased expression levels of the AMPA receptors with similar kinetics. Previous studies have shown that changes in spinal NMDA receptors are involved in the development of bone cancer pain [36]. In bone cancer models pain-related behaviors are accompanied by increased expression of NR2B, an NMDA receptor subunit. Interleukin, IL1β released from glial cells is reported to facilitate bone cancer pain by enhancing phosphorylation of NMDA receptor NR-1 subunit [33]. In hippocampal excitatory synapses, D-serine locally released from astrocytes induces long-term potentiation mediated by NMDAR [39]. Therefore, we hypothesize that chemical mediators released from glial cells may control the amplitude of synaptic responses in animals bearing bone cancer by changing expression levels of NMDA and AMPA receptors and their phosphorylation. However, further experiments will be needed to elucidate the functional mechanisms of glutamate receptor enhancement in bone cancer pain models.

### Plastic changes in spinal sensory pathways from afferent fibers in bone cancer model

In this study, we recorded monosynaptic EPSCs in SG neurons mediated through Aδ and C afferent fibers in control and cancer-bearing mice. Most of the recorded neurons did not receive mono- or polysynaptic inputs from afferent fibers (Table 1). The percentage of neurons receiving monosynaptic inputs was lower in comparison with that in previous studies using rats [27,28]. This difference may be due to different experimental conditions, such as the smaller size of the dorsal root attached to the same thickness of transverse spinal cord slices or species differences. Although the number of SG neurons receiving monosynaptic inputs from Aδ and C fibers was not significantly different between control and cancer-bearing mice, the amplitude of monosynaptic C fiber-evoked EPSCs was significantly increased in cancer-bearing mice, whereas that of monosynaptic Aδ fiber-evoked EPSCs was unchanged (Figure 4). This represents a unique functional alteration in the spinal synaptic transmission compared with plastic changes in other chronic pain models (see below). The magnitude of the increase in amplitude of monosynaptic C fiber-evoked EPSCs is, however, modest, compared with the changes in the amplitude of AMPA-induced currents and focal EPSCs (Figures 3B, D). This discrepancy suggests that in addition to the direct afferent inputs, other synaptic connections to SG neurons are also modified in cancer-bearing mice. The changes could be detected by examining polysynaptic responses: indeed the numbers of polysynaptic inputs to SG neurons mediated through Aδ and C fibers were also increased in cancer-bearing mice (Table 1). The enhanced polysynaptic inputs might be due to increased excitability of spinal excitatory interneurons by increased excitatory synaptic afferent responses as mentioned above. Consistent with our observation that excitatory synaptic inputs are increased, previous studies have also shown that the excitability (firing rates) of dorsal horn neurons in response to sensory stimulation are increased, and their receptive field sizes are enlarged [8,14,40]. Furthermore, in agreement with our finding that the amplitude of monosynaptic inputs from C fibers is increased, previous studies have demonstrated that firing rates of dorsal horn neurons in response to stimuli that excite C fibers are increased in the bone cancer model [8,41]. It has been reported that myelinated and unmyelinated afferent fibers that innervate cancerous femur are damaged [12,42]. As tumors grow, however, tumor-associated inflammatory cells invade the neoplastic tissue and release protons that generate local acidosis [43]. This tumor-induced release of protons is likely to activate acid sensing afferent fibers, in particular C fibers expressing TRPV1 or ASIC channels that innervate the femur. The selective activation of such acid-sensing C fibers may produce the augmented amplitude of the spinal postsynaptic responses in an activity-dependent manner. However, further studies will be needed to clarify how monosynaptic C fiber-evoked response is specifically modified in bone cancer pain model. In other neuropathic pain models (peripheral nerve injury and inflammation models), mono or polysynaptic inputs mediated through Aβ fibers are reported to be increased in the SG [27-29]. In the present study, we could not detect Aβ fiber-mediated EPSCs in SG neurons of both normal and bone cancer-bearing mice. These differences may account for the different mechanisms that generate bone cancer pain and other neuropathic pain.

## Conclusions

The present study demonstrated that bone cancer pain causes an augmentation of excitatory synaptic transmission in the SG over a wide area of spinal lumbar segments whose dermatomes include the hind paw, and a plastic change in afferent synaptic inputs to SG neurons. This spinal sensitization may be one of the underlying mechanisms for the development of chronic bone cancer pain.

## Methods

### Animals

Experiments were conducted using adult male C3H/HeJ mice (20 - 25 g, 4 - 6 weeks of age) purchased from Japan SLC (Hamamatsu, Japan). The mice were housed in a temperature-controlled room (21 ± 1°C) with a 12 h light/dark cycle, and given free access to food and water. The animal experiments were approved by the Sapporo Medical University Animal Care Committee and the committee of the ethics on Animal Experiment, Kyushu University, and were conducted in accordance with the Guidelines of the Japanese Physiological Society and the Ethical Guidance and of the National Institutions of Health, USA. All efforts were made to minimize animal suffering and to reduce the number of animals that were used.

### Bone cancer pain model

Murine sarcoma cells (NCTC 2472; ATCC, Rockville, MD, USA) were maintained in NCTC 135 media containing 10% horse serum (HyClone, Logan, UT, USA) and passaged weekly according to the ATCC recommendation. Implantation of sarcoma cells was performed as previously described [16]. Briefly, mice were anesthetized with isoflurane (3% in 100% oxygen), and a superficial incision was made in the skin overlying the left patella. The patellar ligament was then cut to expose the condyle of the distal femur. A 0.5 mm depression was made using a half-round burr in a pneumatic dental high-speed handpiece to facilitate mechanical retention of the amalgam plug. Then, either 20 μl of α-minimum essential medium (for sham-operated mice) or 20 μl of medium containing 1×10^5 ^sarcoma cells was injected using a 0.25 ml syringe with a 29 gauge needle. To prevent leakage of cells outside the bone, the injection site was closed with dental-grade amalgam, followed by copious irrigation with filtered water. The wound was then closed.

### Behavioral analysis

Quantification of spontaneous flinches was used for assessment of ongoing pain as described previously [44]. The number of spontaneous flinches was recorded during a 2 min observation period. Flinches were defined as holding of the hind paw aloft while not ambulatory. Limb use during spontaneous ambulation was assigned scores on a scale of 0 - 4: 0, complete lack of limb use; 1, relative lack of use of the limb in locomotor activity; 2, limping and guarding behavior; 3, substantial limping; 4, normal use [11,44]. Weight-bearing during standing was scored on a scale of 0 - 3: 0, no weight-bearing (the hind paw on the injected side is always lifted and never touches the floor); 1, touch weight-bearing (the hind paw on the injected side is lifted from the floor but occasionally touches it); 2, partial weight-bearing (partial support of body weight with the hind limb on the injected side); 3, full weight-bearing (full support of 100% of body weight with both hind limbs) [11,44]. In von Frey test, paw withdrawal thresholds in response to probing with von Frey filaments were determined in the manner described by Chaplan et al [45]. Mice were kept in suspended cages with a wire mesh floor, and von Frey filaments were applied perpendicularly to the plantar surface of the ipsilateral paw until it buckled slightly and was held for 3 seconds. A positive response was indicated by a sharp withdrawal of the paw. An initial probe equivalent to 0.008 g was applied and if the response was negative, the stimulus was incrementally increased until a positive response was obtained, then decreased until a negative result was obtained. Mice were allowed to habituate for 30 min before the above all behavioral tests were performed.

### Preparation of spinal cord slices and electrophysiological recordings

The methods used to obtain mouse spinal cord slices with an attached dorsal root were described elsewhere [46,47]. Briefly, the mouse was anesthetized with urethane (1.2 g/kg, i.p.), and a lumbosacral laminectomy (Th11-S1) was performed. The lumbar spinal cord (L1-L6) was removed and placed in preoxygenated cold Krebs solution (1 - 3°C). The mouse was then immediately killed by exsanguination. After cutting all the ventral and dorsal roots near the entry zone, except for the L4 or L5 dorsal root on one side, the pia-arachnoid membrane was removed. The spinal cord was mounted on a microslicer (DSK PRO7, Dosaka, Kyoto, Japan) and then a 600-μm thick transverse slice with the dorsal root was cut. To compare mEPSCs among three groups of SG neurons at different lumbar levels (L2-3, L4 or L5) of spinal cord slices in cancer-bearing mice, we made spinal cord slices with an attached short length (1-2 mm) of rootlet of L2-3, L4 or L5. We first identified the L5 root attached to isolated spinal cord, since the width of L5 rootlet near the entry zone was largest (~2 mm). We then identified the L4 and L2-3 whose rootlet widths were ~1.5 mm and ~1.2 mm, respectively. All the roots were cut and removed except for one short length of the rootlet of L2-3, L4 or L5. After making the spinal cord slices, we identified the lumbar level of slices with the rootlet. The slice was placed on a nylon mesh in the recording chamber (a volume of 0.5 ml), and was completely submerged and perfused at a rate of 12-15 ml/min with Krebs solution equilibrated with 95% O_2 _and 5% CO_2 _at 36 ± 1°C. The Krebs solution contained (in mM): 117 NaCl, 3.6 KCl, 2.5 CaCl_2_, 1.2 MgCl_2_, 1.2 NaH_2_PO_4_, 25 NaHCO_3 _and 11 glucose.

Blind whole-cell patch clamp recordings were made from SG neurons with patch-pipette electrodes having a resistance of 8 - 15 MΩ. Neurons in the SG were identified under a binocular microscope in which the SG could be easily distinguished as a colorless band located in the superficial dorsal horn as shown in adult rats [47,48]. The composition of the patch-pipette solution was as follows (in mM): 135 K-gluconate, 5 KCl, 0.5 CaCl_2_, 2 MgCl_2_, 5 EGTA, 5 HEPES, and 5 ATP (magnesium salt) (pH 7.2, adjusted with KOH, 285 m osmol/l). Cs-based patch-pipette solution composed of 110 Cs_2_SO_4_, 5 tetraethylammonium (TEA), 0.5 CaCl_2_, 2 MgCl_2_, 5 EGTA, 5 HEPES, and 5 ATP (magnesium salt) (pH 7.2, adjusted with CsOH, 285 m osmol/l) was used for recording *N*-methyl-*D*-aspartate (NMDA) receptor mediated responses at a holding potential of +40 mV. Signals were acquired with an Axopatch 200B amplifier (Molecular Devices, California, USA). Currents in the voltage-clamp mode were low-pass filtered at 2-5 kHz and digitized at 333 kHz with an A/D converter (Digidata 1322, Molecular Device). The data were stored and analyzed using Clampex 9.0 software (Molecular Device) and MiniAnalysis software (version 6.0.7, Synaptosoft, Inc., Decatur, USA). Focal EPSCs were evoked at a frequency of 0.2 Hz by stimulation with a monopolar silver wire electrode (diameter: 50 μm), insulated except for the tip, located within 250 μm of the recorded neurons, as reported previously [49]. Root-evoked EPSCs were elicited by stimuli given to the dorsal root at a frequency of 0.2 Hz (unless otherwise mentioned) via a suction electrode. Aδ or C fiber-evoked EPSCs were distinguished on the basis of the conduction velocity of afferent fibers (Aδ, 2-13 m/s; C, < 0.8 m/s) and stimulus threshold (Aδ, 10-60 μA; C, 160-530 μA), as described previously [28]. The Aδ and C responses were considered as monosynaptic in origin when the latency remained constant and there was no failure during stimulation at 20 Hz for the Aδ fiber-evoked EPSCs, and at 2 Hz for the C fiber-evoked EPSCs [28]. The threshold for polysynaptic inputs was sometimes higher than that for monosynaptic inputs. However, the threshold for polysynaptic Aδ fiber-evoked inputs were always lower than those for monosynaptic C fiber-evoked EPSCs. For the Aδ fiber-evoked EPSCs, therefore, we differentiated between mono- or polysynaptic nature with the stimulus intensity which is 1.5 times higher than the threshold and yet is still much lower than that for C fibers. To know whether polysynaptic C fiber inputs are evoked in recorded neurons, we used a supramaximal amplitude of stimulus current (10 mA) for all of the recorded cells.

### Statistical analysis

All numerical data are expressed as mean ± SEM. Statistical significance was determined at the *P *< 0.05 level using unpaired t-test or Mann-Whitney U-test at comparison between 2 groups, and using non-repeated measures ANOVA by post hoc test or Dunnett's test at comparison between 3 groups.

## Abbreviations

AMPA: α-amino-3-hydroxy-5-methyl-4-isoxazolepropionic acid; APV: D-2-amino-5-phosphonovaleric acid; ATCC: American Type Culture Collection; EGTA: ethyleneglycol-bis (b-aminoethyl)-n,n,n',n'-tetraacetic acid; EPSCs: excitatory post synaptic currents; focal AMPAR-EPSCs: focal AMPA receptor-mediated EPSCs; focal NMDAR-EPSCs: focal NMDA receptor-mediated EPSCs; HEPES: 4-(2-hydroxyethyl)-1-piperazineethanesulfonic acid; ATP: adenosine triphosphate; IL 1β: interleukin 1β mEPSCs: miniature EPSCs; CNS: central nervous system; NMDA: *N*-methyl-*D*-aspartate; sEPSCs: spontaneous excitatory post synaptic currents; SG: substantia gelatinosa; TEA: tetraethylammonium; CNQX: 6-cyno-7-nitro-quinoxaline-2,3-dione; TTX: tetrodotoxin

## Competing interests

The authors declare that they have no competing interests.

## Authors' contributions

YY carried out the majority of the experiments and the data analysis. JY and SF participated in producing bone cancer pain model mice and conducted behavioral experiments. DU performed additional experiments. HF, KI, TK, and YY conceptualized the project and formulated the hypothesis and wrote the manuscript. HF, YI and MY designed and directed the experiments. All authors read and approved the final manuscript.
